# Draft Genome Sequence of the Gluten-Hydrolyzing Bacterium *Bacillus subtilis* GS 188, Isolated from Wheat Sourdough

**DOI:** 10.1128/genomeA.00952-17

**Published:** 2017-09-07

**Authors:** B. S. Rashmi, Devaraja Gayathri

**Affiliations:** Department of Microbiology, Davangere University, Shivagangothri, Davangere, Karnataka, India

## Abstract

The draft genome sequence of *Bacillus subtilis* GS 188, a novel spore-forming probiotic bacterium with gluten-hydrolyzing potential, was isolated from wheat sourdough and provides deep insights into the beneficial features of this strain for its use in the preparation of gluten-reduced wheat foods for humans with celiac disease.

## GENOME ANNOUNCEMENT

*Bacillus* spp. are currently of keen interest to the probiotic industries, as they are able to withstand various adverse conditions in the gastrointestinal tract and also in consumer products with indefinite shelf lives (1, 2). Moreover, compared to conventional probiotic lactic acid bacteria, *Bacillus* spp., particularly *B. subtilis*, *B. licheniformis*, *B. clausii*, and *B. coagulans*, have been proven to exhibit potential probiotic attributes (1, 3). Therefore, *Bacillus* sp. probiotics have been proposed as nutritional supplements or novel foods (4). However, data on gluten-hydrolyzing *B. subtilis* probiotics are scarce. Given this, in an earlier study we reported the probiotic potentiality of the gluten-hydrolyzing *B. subtilis* GS 188 (5), and here we report the draft genome sequence of the isolate in order to unravel the genetic blueprint that confers gluten-hydrolyzing potential along with probiotic traits and safe use.

Genomic DNA was isolated as described previously (6), and the paired-end sequencing libraries were prepared using an Illumina TruSeq Nano DNA library preparation kit according to the manufacturer’s instructions. The mean fragment size distribution of the constructed libraries was 525 bp. Further, the libraries were sequenced on the Illumina NextSeq500 platform using 2 × 150-bp chemistry at Eurofins Genomics (India). The raw reads were filtered using Trimmomatic version 0.35 (7). The filtered 6,639,770 paired-end reads with an average GC content of 44.81% were mapped to the *B. subtilis* ASM904v1 reference genome using the Burrows-Wheeler aligner. Then, a consensus sequence comprising 4,022,027 bp was obtained using SAMtools mpileup (8). Further, with the help of bedtools, the genes were extracted from the consensus using the gene coordinates obtained from GenBank.

A total of 4,141 genes were identified from *B. subtilis* GS 188 with maximum and minimum lengths of 16,466 and 41 bp, respectively. Of these genes, 2,527 were annotated using Blast2Go, while gene ontology annotations were determined using the UniprotKB database. Annotation predicted the presence of genes encoding proteins for motility, sporulation, antimicrobial activity, and cell-to-cell interaction for biofilm formation. In addition, the presence of genes for peptidases and extracellular proteases, along with the genes encoding cell membrane-associated proteins of the high-affinity amino acid transporter system, indicated the extensive proteolytic potential of the isolate. Further, to identify the potential involvement of the genes of *B. subtilis* GS 188 in biological pathways, genes were mapped to reference canonical pathways in KEGG. The identified 4,141 genes were provided as input to KEGG-KAAS. KEGG pathway analysis classified 1,944 enriched genes in the KEGG database into 24 functional pathways; of these genes, the highest numbers accounted for carbohydrate metabolism (256), amino acid metabolism (201), membrane transport (180), metabolism of cofactors and vitamins (145), signal transduction (132), and lipid metabolism (67).

The genetic blueprint of *B. subtilis* GS 188 has thrown light on the molecular mechanisms accounting for its probiotic and gluten-hydrolyzing potential, which consequently encourages the safe use of the indigenous isolate as a probiotic.

### Accession number(s).

This whole-genome shotgun project has been deposited in GenBank under the accession no. CP022391. The version described in this paper is the first version.
